# How do women living with HIV experience menopause? Menopausal symptoms, anxiety and depression according to reproductive age in a multicenter cohort

**DOI:** 10.1186/s12905-021-01370-w

**Published:** 2021-05-28

**Authors:** Ines Suarez-García, Belén Alejos, Maria-Jesús Pérez-Elías, Jose-Antonio Iribarren, Asunción Hernando, Margarita Ramírez, María Tasias, Mario Pascual, Inma Jarrin, Victoria Hernando, CoRIS Cohort, Santiago Moreno, Inma Jarrín, David Dalmau, Maria Luisa Navarro, Maria Isabel González, Federico Garcia, Eva Poveda, Jose Antonio Iribarren, Félix Gutiérrez, Rafael Rubio, Francesc Vidal, Juan Berenguer, Juan González, M.Ángeles Muñoz-Fernández, Inmaculada Jarrin, Belén Alejos, Cristina Moreno, Carlos Iniesta, Luis Miguel Garcia Sousa, Nieves Sanz Perez, Marta Rava, Irene Consuegra Fernández, Esperanza Merino, Gema García, Irene Portilla, Iván Agea, Joaquín Portilla, José Sánchez-Payá, Juan Carlos Rodríguez, Lina Gimeno, Livia Giner, Marcos Díez, Melissa Carreres, Sergio Reus, Vicente Boix, Diego Torrús, Ana López Lirola, Dácil García, Felicitas Díaz-Flores, Juan Luis Gómez, María del Mar Alonso, Ricardo Pelazas, Jehovana Hernández, María Remedios Alemán, María Inmaculada Hernández, Víctor Asensi, Eulalia Valle, María Eugenia Rivas Carmenado, Tomás Suárez-Zarracina Secades, Laura Pérez Is, Federico Pulido, Otilia Bisbal, Asunción Hernando, Lourdes Domínguez, David Rial Crestelo, Laura Bermejo, Mireia Santacreu, José Antonio Iribarren, Julio Arrizabalaga, María José Aramburu, Xabier Camino, Francisco Rodríguez-Arrondo, Miguel Ángel von Wichmann, Lidia Pascual Tomé, Miguel Ángel Goenaga, Mª Jesús Bustinduy, Harkaitz Azkune, Maialen Ibarguren, Aitziber Lizardi, Xabier Kortajarena, Mª Pilar Carmona Oyaga, Maitane Umerez Igartua, Mar Masiá, Sergio Padilla, Catalina Robledano, Joan Gregori Colomé, Araceli Adsuar, Rafael Pascual, Marta Fernández, José Alberto García, Xavier Barber, Vanessa Agullo Re, Javier Garcia Abellán, Reyes+ Pascual Pérez, María Roca, Roberto Muga, Arantza Sanvisens, Daniel Fuster, Juan Carlos López Bernaldo de Quirós, Isabel Gutiérrez, Margarita Ramírez, Belén Padilla, Paloma Gijón, Teresa Aldamiz-Echevarría, Francisco Tejerina, Francisco José Parras, Pascual Balsalobre, Cristina Diez, Leire Pérez Latorre, Chiara Fanciulli, Joaquín Peraire, Consuelo Viladés, Sergio Veloso, Montserrat Vargas, Montserrat Olona, Anna Rull, Esther Rodríguez-Gallego, Verónica Alba, Alfonso Javier Castellanos, Miguel López-Dupla, Marta Montero Alonso, José López Aldeguer, Marino Blanes Juliá, María Tasias Pitarch, Iván Castro Hernández, Eva Calabuig Muñoz, Sandra Cuéllar Tovar, Miguel Salavert Lletí, Juan Fernández Navarro, Juan González-Garcia, Francisco Arnalich, José Ramón Arribas, Jose Ignacio Bernardino de la Serna, Carmen Busca, Joanna Cano, Julen Cardiñanos, Juan Miguel Castro, Ana Delgado Hierro, Luis Escosa, Pedro Herranz, Víctor Hontañón, Silvia García-Bujalance, Milagros García López-Hortelano, Alicia González-Baeza, Rosa de Miguel, Maria Luz Martín-Carbonero, Mario Mayoral, Maria Jose Mellado, Rafael Esteban Micán, Rocio Montejano, María Luisa Montes, Victoria Moreno, Ignacio Pérez-Valero, Guadalupe Rúa Cebrián, Berta Rodés, Talia Sainz, Elena Sendagorta, Eulalia Valencia, José Ramón Blanco, José Antonio Oteo, Valvanera Ibarra, Luis Metola, Mercedes Sanz, Laura Pérez-Martínez, Piedad Arazo, Gloria Sampériz, Angels Jaén, Montse Sanmartí, Mireia Cairó, Javier Martinez-Lacasa, Pablo Velli, Roser Font, Marina Martinez, Francesco Aiello, Maria Rivero Marcotegui, Jesús Repáraz, María Gracia Ruiz de Alda, María Teresa de León Cano, Beatriz Pierola Ruiz de Galarreta, María José Amengual, Gemma Navarro, Manel Cervantes Garcia, Sonia Calzado Isbert, Marta Navarro Vilasaro, Ignacio de los Santos, Jesús Sanz Sanz, Ana Salas Aparicio, Cristina Sarria Cepeda, Lucio Garcia-Fraile Fraile, Enrique Martín Gayo, José Luis Casado Osorio, Fernando Dronda Nuñez, Ana Moreno Zamora, Maria Jesús Pérez Elías, Carolina Gutiérrez, Nadia Madrid, Santos del Campo Terrón, Sergio Serrano Villar, Maria Jesús Vivancos Gallego, Javier Martínez Sanz, Usua Anxa Urroz, Tamara Velasco, Enrique Bernal, Alfredo Cano Sanchez, Antonia Alcaraz García, Joaquín Bravo Urbieta, Ángeles Muñoz Perez, Maria Jose Alcaraz, Maria del Carmen Villalba, Federico García, José Hernández Quero, Leopoldo Muñoz Medina, Marta Alvarez, Natalia Chueca, David Vinuesa García, Clara Martinez-Montes, Carlos Guerrero Beltrán, Adolfo de Salazar Gonzalez, Ana Fuentes Lopez, Jorge Del Romero, Montserrat Raposo Utrilla, Carmen Rodríguez, Teresa Puerta, Juan Carlos Carrió, Mar Vera, Juan Ballesteros, Oskar Ayerdi, Antonio Antela, Elena Losada, Melchor Riera, María Peñaranda, Mª Angels Ribas, Antoni A. Campins, Carmen Vidal, Francisco Fanjul, Javier Murillas, Francisco Homar, Helem H. Vilchez, Maria Luisa Martin, Antoni Payeras, Jesús Santos, Cristina Gómez Ayerbe, Isabel Viciana, Rosario Palacios, Carmen Pérez López, Carmen Maria Gonzalez-Domenec, Pompeyo Viciana, Nuria Espinosa, Luis Fernando López-Cortés, Daniel Podzamczer, Arkaitz Imaz, Juan Tiraboschi, Ana Silva, María Saumoy, Paula Prieto, Esteban Ribera, Adrian Curran, Julián Olalla Sierra, Javier Pérez Stachowski, Alfonso del Arco, Javier de la torre, José Luis Prada, José María García de Lomas Guerrero, Onofre Juan Martínez, Francisco Jesús Vera, Lorena Martínez, Josefina García, Begoña Alcaraz, Amaya Jimeno, Ángeles Castro Iglesias, Berta Pernas Souto, Álvaro Mena de Cea, Josefa Muñoz, Miren Zuriñe Zubero, Josu Mirena Baraia-Etxaburu, Sofía Ibarra Ugarte, Oscar Luis Ferrero Beneitez, Josefina López de Munain, Mª Mar Cámara López, Mireia de la Peña, Miriam Lopez, Iñigo Lopez Azkarreta, Carlos Galera, Helena Albendin, Aurora Pérez, Asunción Iborra, Antonio Moreno, Maria Angustias Merlos, Asunción Vidal, Marisa Meca, Concha Amador, Francisco Pasquau, Javier Ena, Concha Benito, Vicenta Fenoll, Concepción Gil Anguita, José Tomás Algado Rabasa, Inés Suárez-García, Eduardo Malmierca, Patricia González-Ruano, Dolores Martín Rodrigo, Mª Pilar Ruiz Seco, Mohamed Omar Mohamed-Balghata, María Amparo Gómez Vidal, Miguel Alberto de Zarraga, Vicente Estrada Pérez, Maria Jesús Téllez Molina, Jorge Vergas García, Juncal Pérez-Somarriba Moreno, Miguel Górgolas, Alfonso Cabello, Beatriz Álvarez, Laura Prieto, José Sanz Moreno, Alberto Arranz Caso, Cristina Hernández Gutiérrez, María Novella Mena, María José Galindo Puerto, Ramón Fernando Vilalta, Ana Ferrer Ribera, Antonio Rivero Román, Antonio Rivero Juárez, Pedro López López, Isabel Machuca Sánchez, Mario Frias Casas, Angela Camacho Espejo, Miguel Cervero Jiménez, Rafael Torres Perea, Juan A. Pineda, Pilar Rincón Mayo, Juan Macías Sanchez, Nicolás Merchante Gutierrez, Luis Miguel Real, Anais Corma Gomez, Marta Fernández Fuertes, Alejandro Gonzalez-Serna, Alexandre Pérez, Manuel Crespo, Luis Morano, Celia Miralles, Antonio Ocampo, Guillermo Pousada

**Affiliations:** 1 Infectious Diseases Group, Department of Internal Medicine, Hospital Universitario Infanta Sofia, FIIB HUIS HHEN, Madrid, Spain; 2 Universidad Europea de Madrid, Madrid, Spain; 3 National Center for Epidemiology, Institute of Health Carlos III, Avda. Monforte de Lemos, 5, 28029 Madrid, Spain; 4 Hospital Universitario Ramón Y Cajal, IRYCIS, Madrid, Spain; 5 Hospital Universitario de Donostia, ISS BioDonostia, San Sebastian, Spain; 6 Instituto de Investigación Hospital Universitario Doce Octubre, Madrid, Spain; 7 Hospital Universitario Gregorio Marañon, Madrid, Spain; 8 Hospital Universitario La Fe, Valencia, Spain; 9 Institute of Health Carlos III, Telemedicine and E-Health Unit, Madrid, Spain

**Keywords:** Menopause, HIV infection, Symptoms, Anxiety, Depression

## Abstract

**Background:**

To estimate the prevalence and severity of menopausal symptoms and anxiety/depression and to assess the differences according to menopausal status among women living with HIV aged 45–60 years from the cohort of Spanish HIV/AIDS Research Network (CoRIS).

**Methods:**

Women were interviewed by phone between September 2017 and December 2018 to determine whether they had experienced menopausal symptoms and anxiety/depression. The Menopause Rating Scale was used to evaluate the prevalence and severity of symptoms related to menopause in three subscales: somatic, psychologic and urogenital; and the 4-item Patient Health Questionnaire was used for anxiety/depression. Logistic regression models were used to estimate odds ratios (ORs) of association between menopausal status, and other potential risk factors, the presence and severity of somatic, psychological and urogenital symptoms and of anxiety/depression.

**Results:**

Of 251 women included, 137 (54.6%) were post-, 70 (27.9%) peri- and 44 (17.5%) pre-menopausal, respectively. Median age of onset menopause was 48 years (IQR 45–50). The proportions of pre-, peri- and post-menopausal women who had experienced any menopausal symptoms were 45.5%, 60.0% and 66.4%, respectively. Both peri- and post-menopause were associated with a higher likelihood of having somatic symptoms (aOR 3.01; 95% CI 1.38–6.55 and 2.63; 1.44–4.81, respectively), while post-menopause increased the likelihood of having psychological (2.16; 1.13–4.14) and urogenital symptoms (2.54; 1.42–4.85). By other hand, post-menopausal women had a statistically significant five-fold increase in the likelihood of presenting severe urogenital symptoms than pre-menopausal women (4.90; 1.74–13.84). No significant differences by menopausal status were found for anxiety/depression. Joint/muscle problems, exhaustion and sleeping disorders were the most commonly reported symptoms among all women. Differences in the prevalences of vaginal dryness (p = 0.002), joint/muscle complaints (p = 0.032), and sweating/flush (p = 0.032) were found among the three groups.

**Conclusions:**

Women living with HIV experienced a wide variety of menopausal symptoms, some of them initiated before women had any menstrual irregularity. We found a higher likelihood of somatic symptoms in peri- and post-menopausal women, while a higher likelihood of psychological and urogenital symptoms was found in post-menopausal women. Most somatic symptoms were of low or moderate severity, probably due to the good clinical and immunological situation of these women.

## Introduction

Life expectancy among people living with HIV has increased since the advent of highly active antiretroviral therapy (HAART) [1] and thus, many women living with HIV will live to experience menopause. In Spain, 15% of all new HIV diagnoses [2], and 29% of all people living with HIV are women [3]. Median age of women living with HIV has increased from 38.0 years in the year 2003 to 48.6 years in 2018 [3]. These data show that an increasing number of women living with HIV will face the transition to menopause.

Hormonal changes that occur during this stage of women’s life can cause symptoms that impair their quality of life [4]. The symptoms related to menopause include hot flushes, vaginal dryness, sleep disturbances, and psychological and cognitive changes, but not all women will develop these symptoms during the transition to menopause, and not all will develop them with the same intensity [5]. Biological, psychosocial and cultural factors may influence the development of menopausal symptoms, and each woman can experience menopause differently [6]. Although these symptoms are usually of mild intensity and self-limiting, disappearing as the woman progresses in the transition to menopause, they can nevertheless interfere with her daily activities and quality of life.

Women living with HIV infection already face a worse quality of life than men living with HIV [7, 8], and menopausal symptoms can impair their physical wellbeing, although existing studies show conflicting data [9–13]. Moreover, most of the published studies have been performed in North America, where the clinical and psychosocial characteristics of women with HIV may differ from those in other European countries [14].

The aim of our study was to investigate the prevalence and severity of menopausal symptoms, and of anxiety and depression in women living with HIV aged 45–60 years from the cohort of the Spanish HIV/AIDS Research Network (CoRIS), and to assess the differences in these prevalences among the pre-, peri- and post-menopausal women.

## Methods

### Study population

We included all cis-women from the Cohort of the Spanish HIV/AIDS Research Network (CoRIS) aged between 45 and 60 years at 30 November 2016. CoRIS is a prospective, open, multicenter cohort of individuals newly diagnosed with HIV, naïve to antiretroviral treatment at cohort entry. By November 2016, 31 centers from 13 Autonomous Regions in the Spanish public healthcare system were participating in CoRIS. The cohort has been described in detail elsewhere [15].

### Questionnaire design and administration

We used a questionnaire similar to that proposed by Tariq et al. for the PRIME Study [16] to collect sociodemographic, women’s health, sexual, medical and HIV-related information.

The Menopause Rating Scale (MRS) was used to assess the presence and severity of menopausal symptoms [17], which has been used among several populations, including women living with HIV [10, 18]. The MRS is a self-reported subjective scale consisting of 11 items arranged in three subscales: somatic symptoms (sweating/flushes, cardiac complaints, sleeping disorders, and joint/muscle complaints); psychological symptoms (feeling depressed, irritable, anxious, and exhausted); and urogenital symptoms (sexual problems, urinary complaints, and vaginal dryness). Each item is graded as 0 (not present), 1 (mild), 2 (moderate), 3 (severe) or 4 (very severe). The MRS total score is the sum of the scores obtained for each subscale. Somatic, psychological and urogenital symptoms were considered to be present if the sum scores for each specific sub-scale was equal to or above 3, 2 and 1, respectively. Symptoms were considered severe if the score was equal to or above 9 in the somatic sub-scale, 7 in the psychological sub-scale and 4 in the urogenital sub-scale. For each sub-scale, each specific symptom was considered to be present if it scored equal to or above 2.

The 4-item Patient Health Questionnaire (PHQ-4) was used to specifically evaluate the current presence of symptoms of anxiety and depression [19, 20]. Women were asked how often they had been bothered over the last two weeks by the following problems: feeling nervous, anxious or on edge; not being able to stop or control worrying; feeling down, depressed or hopeless; and having little interest or pleasure in doing things. Each question was graded as 0 (not at all), 1 (several days), 2 (more than half the days), and 3 (nearly every day). The total score was determined by adding together the scores for each of the 4 items. A total score higher than 3 for the questions “Feeling nervous, anxious or on edge” and “Not being able to stop or control worrying” was identified as anxiety. A total score higher than 3 for the questions “Feeling down, depressed or hopeless” and “Little interest or pleasure in doing things” was identified as depression. Both anxiety and depression were considered to be present if the total score was equal to or above 6.

Social support was evaluated through a 4-item MOS-SSS version [21], ranging from 1 (never) to 5 (all of the time) for each item. “Social support” was classified as “poor” when the score was below 12 points and “normal” if the score was 12 points or higher.

A healthcare provider in each participant center contacted the women who met inclusion criteria to explain the project and to request their participation and written consent. Women who agreed to participate were interviewed by phone between September 2017 and December 2018. Responses were anonymized for the investigators analyzing the data.

### Menopausal status definitions

Menopausal status was self-reported, as, in other similar published studies [9, 11, 22, 23], and was categorized as: pre-menopausal, if a woman had regular menstruation; peri-menopausal, if a woman reported amenorrhea for at least three months in the previous year; and post-menopausal, if a woman had 12 consecutive months of amenorrhea. Women who had a history of hysterectomy and/or bilateral oophorectomy were excluded.

### Statistical analysis

Sociodemographic and clinical characteristics according to menopausal status were summarized using frequency distributions for categorical variables and median and interquartile range (IQR) for continuous ones. The χ^2^ test for independence was used for comparison of categorical variables and the non-parametric Kruskal–Wallis test for comparison of continuous ones.

We described the prevalence and the severity of somatic, psychological and urogenital symptoms, the prevalence of each specific symptom and the prevalence of anxiety and depression, according to menopausal status.

Logistic regression models were used to estimate odds ratios (ORs) of association between menopausal status, and other potential risk factors, the presence and severity of somatic, psychological and urogenital symptoms, and of anxiety and depression. Multivariable models included menopausal status, mode of transmission (heterosexual, injecting drug users (IDU), other), current occupational status (employed, unemployed, others), current partner (no, yes) smoking (never/past, current), drug use (never/sporadic, past/current) and social support (normal, poor). Wald tests were used to derive p values. All statistical analyses were performed using Stata software (version 16.0; Stata Corporation, College Station, TX, USA).

### Ethics

Each patient signed an informed consent to participate in the cohort. The HIV Biobank Ethics’ Committees approved the CoRIS cohort and the Ethics Committee of the Carlos III Institute of Health approved this study.

## Results

The CoRIS database, updated on November 30^th^, 2016, included data from 10,316 individuals, of which 1,681 were women; of these, 528 women were aged between 45 and 60 years. Among the 528 women aged 45–60 years, 277 were excluded for the following reasons: 97 (35.0%) declined to participate in the study, 93 (33.6%) could not be contacted, 36 (13.0%) had a history of hysterectomy and/or bilateral oophorectomy; 20 (7.2%) had died between their last registered visit in the cohort and this study, and 31 (11.2%) were excluded for different reasons (not speaking Spanish, health problems, being transgender, not having a phone). The women who were excluded were more likely to have low educational level (p < 0.001) and to have been born in countries other than Spain or South American countries (p < 0.001) compared to those who were included in the study.

Finally 251 women were included in this study, of which 137 (54.6%) had reached menopause, 70 (27.9%) were peri-menopausal and 44 (17.5%) were pre-menopausal. Among menopausal women, the median age at their final menstrual period was 48 years (IQR 45–50).

The sociodemographic and clinical characteristics according to menopausal status are shown in Table 1.

**Table 1:** Sociodemographic and clinical characteristics of 251 women included, according to menopausal status

		Menopausal status			
	All women	Pre-menopausal	Peri-menopasual	Post-menopausal	p
	251 (100%)	44 (17.5%)	70 (27.9%)	137 (54.6%)	
*Sociodemographic variables*					
Age (years; median –[IQR])	52 (48–55)	46 (45–48)	49 (47–52)	55 (52–57)	**< 0.001**
Country of origin					
Spain	187 (74.5%)	26 (59.1%)	53 (75.7%)	108 (78.8%)	**0.032**
Others	64 (25.5%)	18 (40.9%)	17 (24.3%)	29 (21.2%)	
Educational level					
None or primary	21 (8.4%)	2 (4.5%)	7 (10.0%)	12 (8.8%)	0.574
Secondary or university	230 (91.6%)	42 (95.5%)	63 (90.0%)	125 (91.2%)	
Current occupational status					
Employed	137 (54.6%)	24 (54.5%)	41 (58.6%)	72 (52.6%)	0.525
Unemployed	45 (17.9%)	11 (25.0%)	11 (15.7%)	23 (16.8%)	
Other*	69 (27.5%)	9 (20.5%)	18 (25.7%)	42 (30.7%)	
Current partner and cohabitation status					
Without partner	106 (42.2%)	16 (36.4%)	19 (27.1%)	71 (51.8%)	**0.004**
With partner and living together	108 (43.0%)	24 (54.5%)	35 (50.0%)	49 (35.8%)	
With partner and not living together	37 (14.7%)	4 (9.1%)	16 (22.9%)	17 (12.4%)	
*Clinical variables*					
Body mass index (kg/m^2^)					
< 18.5	15 (6.0%)	3 (6.8%)	2 (2.9%)	10 (7.3%)	0.482
18.5–24.9	136 (54.2%)	23 (52.3%)	39 (55.7%)	74 (54.0%)	
25–30	54 (21.5%)	10 (22.7%)	20 (28.6%)	24 (17.5%)	
> 30	39 (15.5%)	6 (13.6%)	7 (10.0%)	26 (19.0%)	
Unknown	7 (2.8%)	2 (4.5%)	2 (2.9%)	3 (2.2%)	
Body mass index (kg/m2)	23.7 (21.2–28.0)	24.0 (22.0–26.7)	23.8 (21.2–27.7)	23.6 (21.0–28.7)	0.982
Hepatitis B					
Yes	60 (23.9%)	6 (13.6%)	18 (25.7%)	36 (26.3%)	0.335
No	174 (69.3%)	33 (75.0%)	47 (67.1%)	94 (68.6%)	
Unknown	17 (6.8%)	5 (11.4%)	5 (7.1%)	7 (5.1%)	
Hepatitis C					
Yes	60 (23.9%)	6 (13.6%)	16 (22.9%)	38 (27.7%)	0.204
No	179 (71.3%)	34 (77.3%)	50 (71.4%)	95 (69.3%)	
Unknown	12 (4.8%)	4 (9.1%)	4 (5.7%)	4 (2.9%)	
Diabetes mellitus					
Yes	18 (7.2%)	2 (4.5%)	4 (5.7%)	12 (8.8%)	0.549
No	233 (92.8%)	42 (95.5%)	66 (94.3%)	125 (91.2%)	
Osteoporosis					
Yes	37 (14.7%)	0 (0.0%)	7 (10.0%)	30 (21.9%)	**< 0.001**
No	214 (85.3%)	44 (100.0%)	63 (90.0%)	107 (78.1%)	
Smoking					
Current	115 (45.8%)	15 (34.1%)	35 (50.0%)	65 (47.4%)	0.215
Never/past	136 (54.2%)	29 (65.9%)	35 (50.0%)	72 (52.6%)	
Alcohol consumption					
Never/occasional (< 2 times/week)	202 (80.5%)	36 (81.8%)	56 (80.0%)	110 (80.3%)	0.969
Frequent (≥ 2 times/week)	49 (19.5%)	8 (18.2%)	14 (20.0%)	27 (19.7%)	
Drug use					
Never	154 (61.4%)	27 (61.4%)	43 (61.4%)	84 (61.3%)	0.522
Sporadic use	8 (3.2%)	3 (6.8%)	3 (4.3%)	2 (1.5%)	
Regular past use	75 (29.9%)	13 (29.5%)	21 (30.0%)	41 (29.9%)	
Regular current use	14 (5.6%)	1 (2.3%)	3 (4.3%)	10 (7.3%)	
Trouble failing asleep the last two weeks					
Never	137 (54.6%)	28 (63.6%)	44 (62.9%)	65 (47.4%)	0.165
Less than twice a week	29 (11.6%)	5 (11.4%)	6 (8.6%)	18 (13.1%)	
More than 3 times a week	85 (33.9%)	11 (25.0%)	20 (28.6%)	54 (39.4%)	
Social support (MOS-SSS-4item)					
Poor(score < 12)	43 (17.1%)	8 (18.2%)	4 (5.7%)	31 (22.6%)	**0.009**
Normal(score ≥ 12)	208 (82.9%)	36 (81.8%)	66 (94.3%)	106 (77.4%)	
Sexual intercourse during last year					
Yes	159 (63.3%)	33 (75.0%)	56 (80.0%)	70 (51.1%)	**< 0.001**
No	92 (36.7%)	11 (25.0%)	14 (20.0%)	67 (48.9%)	
Age at diagnosis (years; median[IQR])	42 (38–46)	39 (36.5–42)	40 (36–44)	45 (39–49)	**< 0.001**
HIV transmission mode					
Injecting drug user	30 (12.0%)	3 (6.8%)	10 (14.3%)	17 (12.4%)	0.293
Heterosexual	210 (83.7%)	37 (84.1%)	59 (84.3%)	114 (83.2%)	
Others/Unknown	11 (4.4%)	4 (9.1%)	1 (1.4%)	6 (4.4%)	
Time since HIV diagnoses (years)					
< 5	44 (18%)	10 (22.7%)	11 (15.7%)	23 (16.8%)	0.667
5–10	85 (34%)	17 (38.6%)	24 (34.3%)	44 (32.1%)	
> 10	122 (49%)	17 (38.6%)	35 (50.0%)	70 (51.1%)	
Antiretroviral treatment					
Yes	236 (94%)	42 (95.5%)	66 (94.3%)	128 (93.4%)	0.88
No	15 (6%)	2 (4.5%)	4 (5.7%)	9 (6.6%)	
Time with ART (years)					
Median (IQR)	7.5 (4.8–10.5)	6.8 (4.2–10.1)	7.8 (5.2–10.3)	7.5 (4.6–10.6)	0.471
ART adherence					
All pills	222 (94.1%)	39 (92.9%)	60 (90.9%)	123 (96.1%)	0.407
Most of them	11 (4.7%)	3 (7.1%)	4 (6.1%)	4 (3.1%)	
Half of them or less	3 (1.3%)	0 (0.0%)	2 (3.0%)	1 (0.8%)	
AIDS diagnosis					
Yes	52 (20.7%)	7 (15.9%)	14 (20.0%)	31 (22.6%)	0.623
No	199 (79.3%)	37 (84.1%)	59 (84.3%)	106 (77.4%)	
CD4 count at last visit (cells/µl)					
< 200	7 (2.8%)	0 (0.0%)	1 (1.4%)	6 (4.4%)	0.484
200–500	60 (23.9%)	11 (25.0%)	15 (21.4%)	34 (24.8%)	
> 500	184 (73.3%)	33 (75.0%)	54 (77.1%)	97 (70.8%)	
Median (IQR)	720 (491–960)	663 (500–925.5)	719.5 (527–959)	731 (476–1016)	0.929
Viral load at last visit					
Undetectable(≤ 200 copies/µl)	234 (93.2%)	41 (93.2%)	68 (97.1%)	125 (91.2%)	0.279
Detectable(> 200 copies/µl)	17 (6.8%)	3 (6.8%)	2 (2.9%)	12 (8.8%)	
*Women*’*s health variables*					
Age at menarche (years)					
< 13	98 (39%)	16 (36.4%)	28 (40.0%)	54 (39.4%)	0.990
≥ 13	149 (59%)	27 (61.4%)	41 (58.6%)	81 (59.1%)	
Unknown	4 (02%)	1 (2.3%)	1 (1.4%)	2 (1.5%)	
Number of pregnancies					
None	36 (14.3%)	7 (15.9%)	13 (18.6%)	16 (11.7%)	0.344
1 or 2	114 (45.4%)	17 (38.6%)	27 (38.6%)	70 (51.1%)	
≥ 3	101 (40.2%)	20 (45.5%)	30 (42.9%)	51 (37.2%)	

Bold values are statistically significant

*IQR* interquartile range, *ART* antiretroviral therapy, *AIDS* acquired immunodeficiency syndrome

^*^Other: retired, housewife, students

The median age was 46, 49 and 55 years for pre-, peri- and post-menopausal women, respectively (p < 0.001). Pre-menopausal women were more likely to have been born in countries other than Spain (40.9%) compared with peri- and post-menopausal women (24.3% and 21.2%, respectively). Post-menopausal women most frequently had a partner with whom they lived (51.8%) whereas pre- and peri-menopausal most frequently had a partner but they did not live together (54.5% and 50.0%, respectively). Not having a partner was reported by 9.1%, 22.9% and 12.4% of pre-, peri- and post-menopausal women, respectively (p = 0.004). Post-menopausal women reported not having had any sexual intercourse during the last past year and having poor social support more frequently than pre-and peri- menopausal women (48.9% versus 25.0% and 22.0% [p < 0.001], and 22.6% versus 18.2% and 5.7% [p = 0.002], respectively).

There were no differences between pre-, peri- and post-menopausal women regarding the consumption of tobacco, alcohol or drugs, or the prevalence of comorbidities—(hepatitis B, hepatitis C, and diabetes mellitus). However, post-menopausal women were more likely to have osteoporosis than the peri-menopausal ones (21.9% vs 10.0%; p < 0.001) and no pre-menopausal women had osteoporosis.

Regarding the variables related to HIV infection, we did not find significant differences between the three groups. Overall, most of the women (83.7%) had acquired HIV infection through heterosexual practices. Ninety-four percent of the women were receiving antiretroviral therapy (ART), of which 94.1% reported full treatment adherence, 93.2% were virally suppressed, and 73.3% had CD4 count > 500 cells/µl in their last follow-up visit.

### Prevalence of menopausal symptoms, severe menopausal symptoms, and anxiety and depression according to menopausal status

Table 2 shows the number and percentage of women experiencing no, mild, moderate or severe somatic, psychological and urogenital symptoms, according to menopausal status.

**Table 2:** Number and percentage of women experiencing no, mild, moderate or severe somatic, psychological and urogenital symptoms according to menopausal status

	Menopausal status			p value
	Pre-menopausal N = 44	Peri-menopausal N = 70	Post-menopausal N = 137	
*Somatic symptoms [N (%)]*				0.321
No symptoms	29 (65.9%)	28 (40.0%)	57 (41.6%)	
Mild	8 (18.2%)	19 (27.2%)	30 (21.9%)	
Moderate	5 (11.4%)	16 (22.8%)	40 (29.2%)	
Severe	2 (4.5%)	7 (10.0%)	10 (7.3%)	
*Psychological symptoms [N (%)]*				0.073
No symptoms	26 (59.1%)	32 (45.7%)	54 (39.4%)	
Mild	8 (18.2%)	11 (15.7%)	24 (17.5%)	
Moderate	4 (9.1%)	14 (20.0%)	31 (22.6%)	
Severe	6 (13.6%)	13 (18.6%)	28 (20.4%)	
*Urogenital symptoms [N (%)]*				0.017
No symptoms	25 (56.8%)	36 (51.4%)	51 (37.2%)	
Mild	6 (13.6%)	15 (21.4%)	24 (17.5%)	
Moderate	10 (22.7%)	13 (18.6%)	28 (20.4%)	
Severe	3 (6.8%)	6 (8.6%)	34 (24.8%)	

Prevalence of somatic, psychological and urogenital symptoms and of anxiety/depression, as well as the ORs for their association with menopausal status, are shown in Table 3.

**Table 3:** Prevalence of somatic, psychological and urogenital symptoms and of anxiety/depression and OR (95% CI) for its association with menopausal status

	Somatic symptoms			Psychological symptoms			Urogenital symptoms			Anxiety/depression		
		Crude	Adjusted^a^		Crude	Adjusted^a^		Crude	Adjusted^a^		Crude	Adjusted^a^
	N (%)	OR [95% CI]	OR [95% CI]	N (%)	OR [95% CI]	OR [95% CI]	N (%)	OR [95% CI]	OR [95% CI]	N (%)	OR [95% CI]	OR [95% CI]
Pre-menopause (N = 44)	15 (34.1%)	1	1	18 (40.9%)	1	1	19 (43.2%)	1	1	5 (11.4%)	1	1
Peri-menopause (N = 70)	42 (60.0%)	**2.90 [1.37–6.16]**	**3.01 [1.38–6.55]**	38 (54.3%)	1.72 [0.71–4.16]	2.18 [0.87–5.40]	34 (48.6%)	1.24 [0.65–2.4]	1.09 [0.57–2.09]	13 (18.6%)	1.78 [0.69–4.60]	2.33 [0.69–7.85]
Post-menopause (N = 137)	80 (58.4%)	**2.71 [1.57–4.69]**	**2.63 [1.44–4.81]**	83 (60.6%)	**2.22 [1.21–4.06]**	**2.16 [1.13–4.14]**	86 (62.8%)	**2.22 [1.26–3.90]**	**2.54 [1.42–4.85]**	30 (21.9%)	2.19 [0.88–5.41]	1.66 [0.48–5.67]
p value	**0.011**			0.072			**0.030**			0.300		

Bold values are statistically significant

OR: Odds Ratio from logistic regression for the association between menopausal status at the presence of menopausal symptoms in each MRS domain and of anxiety/depression

^a^Adjusted for mode of transmission, current occupational status, smoking, drug use, social support, current partner

Results from multivariable analyses showed that post-menopausal women had twice the likelihood of presenting all somatic (OR 2.63; 95% CI 1.44–4.81), psychological (2.16; 1.13–4.14) and urogenital symptoms (2.54; 1.42–4.85) than pre-menopausal women. Peri-menopausal women had a three-fold increase in the likelihood for developing somatic symptoms (OR: 3.01; 95% CI 1.38–6.55) than pre-menopausal women, but no significant differences between pre- and peri-menopausal women were found for psychological or urogenital symptoms. Besides menopausal status and after adjusting for it, other factors that were significantly associated with a higher likelihood of presenting menopausal symptoms were being a current smoker for somatic symptoms (OR 1.90; 95% CI 0.95–3.82), being unemployed as well as having a low social support for psychological symptoms (OR 3.30; 95% CI 1.70–6.42 and OR 4.81; 95% CI1.86–12.46, respectively) and having a current partner for urogenital symptoms (OR: 2.99; 95% CI 1.82–4.390).

We did not find any significant difference in the prevalence of anxiety and depression according to menopausal status (Table 3).

Prevalence of severe somatic, psychological and urogenital symptoms and OR for their association with menopausal status are shown in Table 4. Results from multivariable analyses showed that post-menopausal women had a statistically significant five-fold increase in the likelihood of presenting severe urogenital symptoms than pre-menopausal women (OR 4.90; 95% CI 1.74–13.84). Other factors that increased the likelihood of presenting severe menopausal symptoms, after adjustment for menopausal status, were the current or past use of drugs for both psychological and urogenital symptoms (OR 2.78; 1.23–6.28 and 3.74; 1.78–7.87, respectively), having a low social support for psychological symptoms (3.22; 1.43–7.26) and being unemployed for somatic symptoms (OR 2.48; 95% CI 1.01–6.12).

**Table 4:** Prevalence of severe somatic, psychological and urogenital symptoms and OR (95% CI) for its association with menopausal status

	Severe somatic symptoms			Severe psychological symptoms			Severe urogenital symptoms		
		Crude	Adjusted^a^		Crude	Adjusted^a^		Crude	Adjusted^a^
	N (%)	OR [95% CI]	OR [95% CI]	N (%)	OR [95% CI]	OR [95% CI]	N (%)	OR [95% CI]	OR [95% CI]
Pre-menopause (N = 44)	2 (4.5%)	1	1	6 (13.6%)	1	1	3 (6.8%)	1	1
Peri-menopause (N = 70)	7 (10.0%)	2.33 [0.46–11.82]	3.79 [0.79–18.24]	13 (18.6%)	1.44 [0.54–3.89]	2.31 [0.79–6.80]	6 (8.6%)	1.28 [0.38–5.22]	1.00 [0.27–3.74]
Post-menopause (N = 137)	10 (7.3%)	1.65 [0.31–8.82]	2.35 [0.45–12.86]	28 (20.4%)	1.63 [0.54–4.88]	1.96 [0.47–8.08]	34 (24.8%)	**4.51 [1.42–14.3]**	**4.90 [1.74–13.84]**
p value	0.554			0.602			**0.002**		

Bold values are statistically significant

OR: Odds Ratio from logistic regression for the association between menopausal status and the presence of severe menopausal symptoms in each MRS domain

^a^Adjusted for mode of transmission, current occupational status, smoking, drug use, social support, current partner

**Fig. 1:**
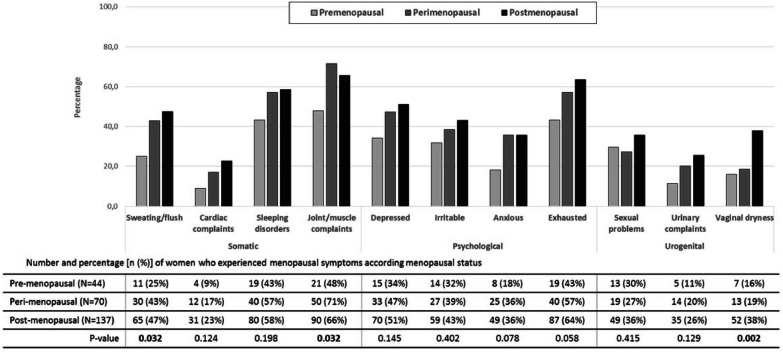
Percentage of menopausal symptoms for each specific domain (psychological, somatic and urogenital) according to menopausal status

The prevalence of each specific symptom in each group is shown in Fig. 1. Overall, the five most commonly reported menopause symptoms were joint/muscle complaints (64%; 95% CI 58–70%), feeling exhausted (58%; 52–64%), sleeping disorders (55%; 49–62%), feeling depressed (47%; 41–53%), and sweating/flush (42%; 36–49%). With the exception of joint and muscle complaints, which were reported more frequently by peri-menopausal women, all the other symptoms were reported more frequently by post-menopausal women, although the differences between the three groups were statistically significant only for sweating and flushes, and vaginal dryness (Fig. 1).

The women were also interviewed about their knowledge and use of selected medications to treat menopausal symptoms. Only 15.9% of pre-, 22.9% of peri- and 27.0% (p = 0.314) of post-menopausal women were aware of hormone replacement therapy (HRT) for menopausal symptoms. The use of HRT was infrequent among these women: four post-menopausal women had received HRT in the past and one pre- and one post-menopausal women were currently using it. These two patients both reported menopausal symptoms in the psychological and urogenital subscales.

Regarding other therapies, 13.6%, 22.9% and 29.9% (p = 0.084) of pre-, peri- and post-menopausal women, respectively, had used non-hormonal lubricants for vaginal dryness. The use of vaginal lubricants was higher among women who experienced moderate or severe urogenital symptoms than among those who had no or mild urogenital symptoms (33.1% versus 15.2%; p = 0.001). Alternative medicines were used to treat menopausal symptoms by 4.6%, 15.7% and 21.9% of pre-, peri- and post-menopausal women, respectively (p = 0.027). Women who had moderate or severe symptoms in the psychological and somatic domains used alternative medicines more frequently than those who had no or mild symptoms in these subscales (23.0% versus 9.8%; p = 0.006 and 23.4% versus 9.6%; p = 0.004, respectively).

## Discussion

In this study we have evaluated the prevalence of somatic, psychological and urogenital symptoms and of anxiety/depression, and its association with menopausal status in women living with HIV from a national cohort. Compared to pre-menopausal women, peri- and post-menopausal women were more likely to experience any somatic symptoms, and post-menopausal women were more likely to experience any psychological and urogenital symptoms. Furthermore, post-menopausal women were more likely to experience severe urogenital symptoms than pre-menopausal women. However, when we evaluated specifically anxiety and depression, we did not find a significant association with menopausal status.

The sample included mostly Spanish women with medium or high educational level, and half of them were currently employed. The vast majority were receiving antiretroviral treatment and had a good clinical and immunological situation. The sociodemographic and clinical variables among pre-, peri- and post-menopausal women were similar.

Being recently diagnosed with HIV is one of the inclusion criteria for the CoRIS cohort, and therefore peri- and post-menopausal women were diagnosed at an older age than premenopausal ones; the three groups had similar times since HIV diagnosis and since ART initiation.

The age at menopause in women living with HIV varies widely among different published studies. Some studies have indicated an earlier onset of menopause among these women compared to HIV-negative ones [9, 24, 25], while others observed a similar age of onset in both groups [26, 27]. In our study, the age of menopause was 48 years, which is similar to that described in the general Spanish population [28].

The clinical profile of women living with HIV infected has a great influence in the onset of menopause, which is influenced by factors such as tobacco and drug use, or low weight. Severe immunodeficiency can affect the length of menstrual cycle and has been associated with early menopause [29]. The proportion of women who used drugs in our cohort was lower than other studies [25], although almost half of women in our study were smokers. The women in our study had a good immunological status, as most of them were receiving ART and the median last CD4 T-cell count was over 700 cells/µl, with 3% having a CD4 T-cell count below 200 cells/µl. Since severe immunodeficiency has been associated with early menopause, starting ART early [30] could improve not only the clinical and immunological situation of women with HIV infection, but it could also lower the risk of an early onset of menopause and its consequences.

It is estimated that 85% of women in Western societies experience menopausal symptoms during years in the menopausal transition. The most common symptoms are hot flushes and night sweats [5, 31], but women can also experience sleep disturbances, fatigue, irritability, decreased concentration, lack of memory and depression [32]. Women who have had a previous mood disorder are more likely to develop a depression during the menopausal transition [33, 34].

More than half of women analyzed experienced menopausal symptoms. Although around a fifth of them experienced severe urogenital and psychological symptoms, the severity of the somatic symptoms was mild or moderate among most women, as found in other studies [18, 35]. Although several other studies showed that women living with HIV experienced more severe somatic menopausal symptoms than the general population [9, 10], this could be explained because the women included in these studies had multiple vulnerabilities (use of drugs, homelessness, violence and mental health problems) which can increase the severity of menopausal symptoms. In our study, the current or past use of drugs was associated with a higher likelihood of experiencing severe psychological and urogenital symptoms.

Vasomotor symptoms (hot flushes and night sweats) are the most frequent symptoms among women during menopause [5, 36]. Sociodemographic and cultural factors, and ethnicity [37], have been shown to be associated with the prevalence of hot flushes. In our study, 25.0%, 42.9% and 47.4% of pre-, peri- and post-menopausal women, respectively, experienced vasomotor symptoms. Mood disorders have been shown to be associated with vasomotor symptoms [38], and several studies have found an association between hot flushes and depressive symptoms in both HIV-infected and non-HIV infected women [12, 39]. Smoking has also been associated with the presence of hot flushes among HIV-infected [39] and non HIV-infected women [40] and also with depression in HIV-infected individuals [41]. In this study half of peri- and post-menopausal women and 34.1% of pre-menopausal ones were smokers, and smoking was associated with a higher likelihood of presenting somatic symptoms. Social support is an important determinant of mental health and quality of life among HIV-infected women [42]. In our study, women reporting low social support were more likely to experience any and severe psychological symptoms. The prevalence of anxiety and depression did not show statistically significant differences according to menopausal status, but women who self-reported poor social support and who did not have a current partner were significantly more likely to have anxiety/depression compared to those with normal social support or who had a current partner.

Unemployment has been associated with an increased likelihood of depressive symptoms [11, 12]. In our study, being unemployed was associated with a higher prevalence of psychological and severe somatic symptoms, and other employment situation different than being unemployed or having a current employment (such as being retired or being a housewife), was associated with the presence of both somatic and psychological symptoms.

It is worth noting that although post-menopausal women reported psychological symptoms more frequently than pre-menopausal ones as assessed by the MRS, anxiety/depression was not significantly associated with menopausal status when using the PHQ-4 scale. This is probably due to differences in the symptoms that both methods (MRS and PHQ-4) asses, as the MRS includes the symptoms of feeling irritable and exhausted, which were frequent among post-menopausal women.

Menopausal symptoms may undermine ART adherence [18, 43]. In this study, women had good level of adherence and a good immunological status. The percentage of women who took all their pills was high and we did not find differences in the adherence to ART among pre-, peri- and post-menopausal women, nor differences in the adherence according to presence of menopausal symptoms and anxiety/depression (data not shown).

Menopausal symptoms are dependent on several biological, social and cultural factors, and due to the very scarce information in the literature, it is unclear whether women with HIV experience menopause differently. This study adds to the knowledge on menopausal symptoms among women living with HIV, in order to inform clinicians and help them improve care of women with HIV during the menopausal transition. BHIVA guidelines recommend proactive assessment of menopausal symptoms on women aged > 45 years [44]. Given that women with HIV more frequently have insufficient knowledge about menopausal symptoms and what to expect during the menopausal transition [6], it is important that clinicians are aware of these symptoms, how they are expressed among women with HIV and the factors associated with their occurrence in order to give them the necessary information.

The use of HRT was very low among these women, as has been described in other studies among women with HIV infection [27, 45], and also among Spanish women from the general population [28]. So far, European guidelines do not provide specific recommendations about hormonal replacement therapy for menopause [46] and Spanish treatment guidelines for women with HIV infection only recommend it when the benefit outweighs the risk [47]. Conversely, British HIV association guidelines for the sexual and reproductive health of people living with HIV recommend that women living with HIV receive HRT as the HIV-negative population [44]. It is worth nothing that a fifth of the postmenopausal women were using alternative medicines to treat menopausal symptoms: this should be taken into account by clinicians as alternative medicine use is frequently not reported by patients and it can cause drug interactions with ART.

Our study has several limitations. We had no data on hormonal levels, such as follicle stimulating hormone (FSH), that would allow a better definition of menopausal status; therefore, the menopausal status was based on self-report about menstrual cycles, as used by other studies [9, 11, 13, 24]. The MRS has been not validated specifically in women living with HIV, however it has been used in several studies carried out among these women and in different settings. Also, the low number of women in our cohort, reflecting the low proportion of women among people living with HIV in Spain [2], is another limitation: this can limit the power to detect differences between the study groups. Also, the women who were not included in the study had lower education level suggesting a lower socioeconomic status, and were originary from countries other than Spain and Latin America, which might limit the generalizability of our findings.

In conclusion, in our study women living with HIV aged 45–60 years experienced a wide variety of menopausal symptoms, and some of the symptoms were initiated before women had any menstrual irregularity. We found a high risk of somatic symptoms in peri- and post-menopausal women, and a high risk of psychological and urogenital symptoms in post-menopausal ones. Most somatic symptoms were of low or moderate severity, probably due to the good clinical and immunological situation of these women. More studies that address needs of women at this stage of life are necessary to identify specific gender gaps.

## Data Availability

Data are available from the corresponding author upon reasonable request and with permission of the Spanish AIDS Research Network.

## Abbreviations

HIV: Human immunodeficiency virus
AIDS: Acquired immunodeficiency syndrome
MRS: Menopause Rating Scale
PHQ-4: Patient Health Questionnaires 4 items
aOR: Adjusted odds ratio
HAART: Highly active antiretroviral therapy
IQR: Interquartile range
IDU: Injecting drug user
ART: Antiretroviral therapy
HRT: Hormone replacement therapy
FSH: Follicle stimulating hormone
